# Outcome effectiveness of the severe sepsis resuscitation bundle with addition of lactate clearance as a bundle item: a multi-national evaluation

**DOI:** 10.1186/cc10469

**Published:** 2011-09-27

**Authors:** H Bryant Nguyen, Win Sen Kuan, Michael Batech, Pinak Shrikhande, Malcolm Mahadevan, Chih-Huang Li, Sumit Ray, Anna Dengel

**Affiliations:** 1 Department of Emergency Medicine, Loma Linda University, 11234 Anderson Street, Loma Linda, CA 92354, USA; 2 Department of Medicine, Division of Pulmonary and Critical Care, Loma Linda University, 11234 Anderson Street, Loma Linda, CA 92354, USA; 3 Department of Emergency Medicine, National University Health System, Street 5 Lower Kent Ridge Road, Singapore 119074, Singapore; 4 Department of Epidemiology and Biostatistics, Loma Linda University, 24951 North Circle Drive, Nichol Hall 1708, Loma Linda, CA 92350, USA; 5 Department of Critical Care Medicine, Fortis Flt. Lt. Rajan Dhall Hospital, Sector B, Pocket-1, Vasant Kunj, New Delhi 110070, India; 6 Department of Emergency Medicine, Chang-Gung Memorial Hospital, Linkou Medical Center, No. 5, Fu-Shing Street, Taoyuan 333, Taiwan, ROC; 7 Graduate Institute of Clinical Medical Sciences, College of Medicine, Chang-Gung University, 259 Wen-Hwa 1st Road, Kwei-Shan, Taoyuan 333, Taiwan, ROC; 8 Department of Critical Care & Emergency Medicine, Sir Gangaram Hospital, Rajinder Nagar, New Delhi 110060, India; 9 Department of Medicine, Loma Linda University, 11234 Anderson Street, Loma Linda, CA 92354, USA

## Abstract

**Introduction:**

Implementation of the Surviving Sepsis Campaign (SSC) guidelines has been associated with improved outcome in patients with severe sepsis. Resolution of lactate elevations or lactate clearance has also been shown to be associated with outcome. The purpose of the present study was to examine the compliance and effectiveness of the SSC resuscitation bundle with the addition of lactate clearance.

**Methods:**

This was a prospective cohort study over 18 months in eight tertiary-care medical centers in Asia, enrolling adult patients meeting criteria for the SSC resuscitation bundle in the emergency department. Compliance and outcome results of a multi-disciplinary program to implement the Primary SSC Bundle with the addition of lactate clearance (Modified SSC Bundle) were examined. The implementation period was divided into quartiles, including baseline, education and four quality improvement phases.

**Results:**

A total of 556 patients were enrolled, with median (25th to 75th percentile) age 63 (50 to 74) years, lactate 4.1 (2.2 to 6.3) mmol/l, central venous pressure 10 (7 to 13) mmHg, mean arterial pressure (MAP) 70 (56 to 86) mmHg, and central venous oxygen saturation 77 (69 to 82)%. Completion of the Primary SSC Bundle over the six quartiles was 13.3, 26.9, 37.5, 45.9, 48.8, and 54.5%, respectively (*P *<0.01). The Modified SSC Bundle was completed in 10.2, 23.1, 31.7, 40.0, 42.5, and 43.6% patients, respectively (*P *<0.01). The ratio of the relative risk of death reduction for the Modified SSC Bundle compared with the Primary SSC Bundle was 1.94 (95% confidence interval = 1.45 to 39.1). Logistic regression modeling showed that the bundle items of fluid bolus given, achieve MAP >65 mmHg by 6 hours, and lactate clearance were independently associated with decreased mortality - having odds ratios (95% confidence intervals) 0.47 (0.23 to 0.96), 0.20 (0.07 to 0.55), and 0.32 (0.19 to 0.55), respectively.

**Conclusions:**

The addition of lactate clearance to the SSC resuscitation bundle is associated with improved mortality. In our study patient population with optimized baseline central venous pressure and central venous oxygen saturation, the bundle items of fluid bolus administration, achieving MAP >65 mmHg, and lactate clearance were independent predictors of outcome.

## Introduction

Despite many advances in our understanding and treatments, epidemiologic studies across the globe have shown an increasing incidence of severe sepsis with a continued high mortality rate [1-3]. As a result, the international Surviving Sepsis Campaign (SSC) developed guidelines to improve the standard of care for severe sepsis. Such guidelines advocate early recognition, broad-spectrum antibiotics, hemodynamic optimization guided by invasive monitoring, corticosteroid, recombinant human activated protein C, and lung protective strategies in the form of the severe sepsis resuscitation and management bundles [4]. While many studies have examined the effectiveness of the bundles only in nonrandomized manners, their successful implementation in America and Europe has been shown to be cost-effective and associated with decreased mortality [5-7].

Crucial to implementing the guidelines and the severe sepsis bundles is the utilization of lactate as an indicator of global organ hypoperfusion and shock. While the etiology of lactate elevations in severe sepsis is debatable, it is well accepted as a resuscitation endpoint, an indicator of severity, and a predictor of short-term and long-term mortality [8-10]. Additionally, persistent elevation of lactate has been shown to be associated with poor outcome, such that a decrease in lactate (or lactate clearance) during resuscitation is an independent predictor for improved mortality [11-13].

Our study was a quality improvement effort in the implementation of the SSC severe sepsis resuscitation bundle in Asia, with the additional aim to examine the effect of lactate clearance as a bundle item. Our hypothesis was that completion of the bundle with addition of lactate clearance will further decrease mortality compared with completion of the bundle without lactate clearance.

## Materials and methods

### Design and setting

This was a multi-national prospective quality improvement cohort study performed in eight tertiary-care medical centers in China, India, Korea, Singapore, and Taiwan. The study was conducted at all participating centers over an 18-month period starting on 1 July 2008 and ending on 31 December 2009. Participating hospitals implemented the severe sepsis resuscitation bundle as recommended by the SSC guidelines to improve the standard of care, with the additional goal of lactate clearance [4,14]. The implementation period was divided into 3-month phases: baseline, education, and quality improvement phases 1 to 4. The Institution Review Board of the participating centers approved the study and waived the need for patient's written consent. Patient data gathered during the course of the study were entered into an Institution Review Board-approved database at the study organization center, Loma Linda University, CA, USA.

### Bundle implementation and lactate clearance

Based on existing hospital staffing and resources, three hospitals adopted a sepsis team model of implementation championed by intensivists. The severe sepsis resuscitation bundle was initiated in the emergency department (ED) and completed in the ICU. Patient care was delivered by a focused multi-disciplinary team led by the attending intensivist, similar to the well-accepted trauma team model. The other five participating hospitals implemented the bundle via a nonteam model, which was championed solely by the ED, with initiation and completion of the bundle by ED physicians and nurses as part of ED standard care.

Delivery of the bundle included lactate measurement, blood cultures obtained prior to antibiotics, early antibiotic administration, hemodynamic monitoring and support, and mechanical ventilation per guidelines [4]. Hemodynamic monitoring was achieved by central venous catheter insertion for continuous monitoring of central venous pressure (CVP) and central venous oxygen saturation (ScvO_2_). Intra-arterial blood pressure monitoring with insertion of a radial or femoral arterial catheter was preferred, especially if patients were on a vasoactive agent. Hemodynamic support included the use of crystalloid fluids, vasoactive and inotropic agents, and packed red blood cell transfusion as appropriate to target optimal CVP, mean arterial pressure (MAP), and ScvO_2 _as previously defined by the early goal-directed therapy (EGDT) protocol [15]. Corticosteroid, glucose control, and lung-protective strategies were provided at the discretion of the treating physicians, and were not considered part of the resuscitation bundle. Recombinant human activated protein C was not available at the participating hospitals.

During the study period, bundle completion was monitored for compliance in each of the implementation phases. A physician champion at each of the participating hospitals completed weekly audits of the bundle implementation. A bundle compliance checklist was completed for each consecutive patient meeting enrollment criteria for the study (see below). Other information recorded for each patient included demographics, laboratories, Acute Physiology and Chronic Health Evaluation (APACHE) II score, vital signs, hemodynamic measurements, source of infection, therapeutic interventions up to 72 hours including ED and ICU lengths of stay, and in-hospital mortality.

Completion of the Primary SSC Bundle included all of the following items: lactate measured; blood cultures before antibiotics; antibiotics administered by 3 hours; fluid bolus given; achieve CVP >8 mmHg by 6 hours; achieve MAP >65 mmHg by 6 hours; and achieve ScvO_2 _>70% by 6 hours (Table 1). Completion of the Modified SSC Bundle was defined as completion of the Primary SSC Bundle and lactate clearance. Lactate clearance was defined as any decrease in lactate within 12 hours from baseline, or an initial lactate <2.0 mmol/l [12,16].

**Table 1:** Surviving Sepsis Campaign severe sepsis resuscitation bundle

Primary SSC Bundle	1. Lactate measured
	2. Blood cultures before antibiotics
	3. Antibiotics administered by 3 hours
	4. Fluid bolus given
	5. Achieve CVP >8 mmHg by 6 hours
	6. Achieve MAP >65 mmHg by 6 hours
	7. Achieve ScvO_2 _>70% by 6 hours
Modified SSC Bundle	Primary SSC Bundle + Lactate clearance

The Primary Surviving Sepsis Campaign (SSC) Bundle includes seven items. The Modified SSC Bundle includes the Primary SSC Bundle and lactate clearance. Lactate clearance is defined as any decrease in lactate within 12 hours from baseline, or an initial lactate <2.0 mmol/l. CVP, central venous pressure; MAP, mean arterial pressure; ScvO_2_, central venous oxygen saturation.

### Patient selection

Adult patients over 18 years of age and meeting criteria for initiating the severe sepsis resuscitation bundle in the ED were considered for enrollment during the weekly audit of bundle implementation. The inclusion criteria were: two of four systemic inflammatory response syndrome criteria; suspected or confirmed infection; and systolic blood pressure <90 mmHg after a 1 liter crystalloid fluid bolus, lactate ≥4 mmol/l, or two or more organ dysfunctions. The systemic inflammatory response syndrome criteria included: temperature >38.3°C or <36°C; heart rate >90 beats per minute; respiratory rate >20 breaths per minute or arterial partial pressure of carbon dioxide <32 mmHg; or a white blood cell count >12, 000 per mm^3 ^or <4, 000 per mm^3^, or bands greater than 10%. Organ dysfunction was defined as: creatinine >2.0 mg/dl or an increase >0.5 mg/dl; PaO_2_/FiO_2 _<300; platelet count <100, 000/μl; International Normalized Ratio >1.5; partial thromboplastin time >60 seconds; or total bilirubin >2.0 mg/dl.

Patients with trauma, active seizures, acute pulmonary edema due to heart failure, a need for emergent surgery, active bleeding, acute stroke, or a do-not-resuscitate order were excluded from the study.

### Outcome measures

The primary measures were compliance and in-hospital mortality associated with completion of the Primary SSC Bundle and the Modified SSC Bundle. Secondary measures included bundle items independently associated with in-hospital mortality.

### Statistical analysis

Univariate analyses were conducted on patient demographics, laboratories, hemodynamics, and therapies provided to the patient to assess normality and to test the assumptions of the parametric and nonparametric statistical tests conducted. Taken into consideration were the following: age, gender, APACHE II score, care team model, bundle completion, mechanical ventilation, therapies in either the ED or ICU, length of stay in the ED, ICU, and hospital, hemodynamic characteristics, laboratory measurements, and infection source. The best measure of central tendency and the corresponding measure of variation for each of these factors stratified by lactate clearance status were determined, reported, and tested for differences using Pearson's chi-square test, Wilcoxon's sum-rank test, or Student's two-sample *t *test as appropriate.

Trends in quarterly compliance to the bundle implementation for the Primary and Modified SSC Bundles were assessed using analysis of variance for completion of the individual bundle items over each of the six quartiles. Comparisons of compliance to the Primary and Modified SSC Bundles were made using Pearson's chi-square test.

Multivariable logistic regression analysis was performed for each of eight different models that sequentially included another bundle item into a base model. The base model included only the measurement of serum lactate (lactate measured), adjusted for the effects of age, gender, APACHE II score, and mechanical ventilation status. The -2 log-likelihood for the model including all covariates was reported and used to compare models with each sequentially included covariate. The corresponding chi-square *P *value for the difference in -2 log-likelihood identified models that significantly improved with the inclusion of the new covariate. A Bonferroni correction was performed to account for multiple comparisons.

Statistical significance was defined at *P *<0.05. All analyses were performed using SAS version 9.2 (SAS Institute Inc., Cary, NC, USA).

## Results

Five-hundred and fifty-six patients were enrolled in the study, with median (25th to 75th percentile) age 63 (50 to 74) years, APACHE II score 22 (16 to 27), and lactate 4.1 (2.2 to 6.3) mmol/l (Table 2). Patients had a median (25th to 75th percentile) length of stay of 5 (3 to 9) hours in the ED and 4 (2 to 8) days in the ICU, and a total hospital stay of 9 (4 to 16) days. A total of 67.1% of patients were in septic shock on presentation, and 53.1% required mechanical ventilation within 72 hours. Baseline (25th to 75th percentile) CVP, MAP, and ScvO_2 _values were 10 (7 to 13) mmHg, 70 (56 to 86) mmHg, and 77 (69 to 82)%, respectively. Pneumonia (41.9%) was the most common source of infection. In-hospital mortality was 29.9%.

**Table 2:** Patient characteristics during the entire study period, comparing lactate clearance with no lactate clearance

	All patients (*n *= 556)	Lactate clearance (*n *= 331)	No lactate clearance (*n *= 225)	*P *value
Age (years)	63 (50 to 74)	61 (50 to 71)	65 (52 to 78)	**<0.01**
Gender				
Female	226 (40.6%)	133 (40.2%)	93 (41.3%)	0.79
Male	330 (59.4%)	198 (59.8%)	132 (58.7%)	
Septic shock	373 (67.1%)	232 (70.1%)	141 (62.7%)	0.07
APACHE II score	22 (16 to 27)	19 (14 to 25)	25 (20 to 30)	**<0.01**
APACHE II predicted mortality (%)	41.6 (20.4 to 59.7)	34.8 (20.4 to 56.1)	52.5 (31.5 to 72.6)	**<0.01**
In-hospital mortality	166 (29.9%)	68 (20.5%)	98 (43.6%)	**<0.01**
Primary SSC Bundle item completion	192 (34.5%)	174 (52.6%)	18 (8.0%)	**<0.01**
Lactate measured	488 (87.8%)	331 (100.0%)	157 (70.4%)	**<0.01**
Blood cultures before antibiotics	514 (92.4%)	313 (94.6%)	201 (89.3%)	**0.02**
Antibiotics administered by 3 hours	537 (96.6%)	324 (97.9%)	213 (94.7%)	**0.04**
Fluid bolus given	506 (91.0%)	310 (93.7%)	196 (87.1%)	**0.01**
Achieve CVP >8 mmHg by 6 hours	319 (57.4%)	232 (70.1%)	87 (38.7%)	**<0.01**
Achieve MAP >65 mmHg by 6 hours	530 (95.3%)	326 (98.5%)	204 (91.5%)	**<0.01**
Achieve ScvO_2 _>70% by 6 hours	250 (45.0%)	213 (64.4%)	37 (16.4%)	**<0.01**
Care team model				
Team	190 (34.2%)	164 (49.6%)	26 (11.6%)	**<0.01**
Nonteam	366 (65.8%)	167 (50.5%)	199 (88.4%)	
Mechanical ventilation	295 (53.1%)	143 (43.2%)	152 (67.6%)	**<0.01**
Therapies in the ED				
Amount of fluids (ml)	1, 500 (1, 000 to 2, 550)	1, 533 (1, 000 to 3, 000)	1, 350 (850 to 2, 300)	**<0.01**
Vasopressor use^a^	306 (55.0%)	188 (56.8%)	118 (52.4%)	0.31
Inotrope use^b^	222 (39.9%)	134 (43.7%)	88 (40.6%)	0.48
Transfusion use	21 (3.8%)	12 (3.6%)	9 (4.0%)	0.82
Therapies in the ICU				
Amount of fluids (ml)	6, 268 (3, 800 to 10, 200)	7, 943 (5, 200 to 11, 345)	4, 596 (3, 060 to 7, 100)	**<0.01**
Vasopressor use^a^	383 (68.9%)	223 (67.4%)	160 (71.1%)	0.35
Inotrope use^b^	251 (45.1%)	141 (44.5%)	110 (49.8%)	0.23
Transfusion use	116 (20.9%)	65 (19.6%)	51 (22.7%)	0.39
Length of stay				
ED (hours)	5 (3 to 9)	4 (3 to 6)	6 (4 to 15)	**<0.01**
ICU (days)	4 (2 to 8)	4 (2 to 7)	4 (1 to 9)	0.53
Hospital (days)	9 (4 to 16)	9 (5 to 16)	9 (3 to 17)	0.07
Hemodynamics				
Baseline CVP (mmHg)	10 (7 to 13)	9 (7 to 12)	11 (6 to 15)	**0.04**
Six-hour CVP (mmHg)	10 (8 to 14)	10 (8 to 13)	10 (7 to 15)	0.20
Baseline MAP (mmHg)	70 (56 to 86)	66 (56 to 83)	73 (57 to 90)	**0.01**
Six-hour MAP (mmHg)	78 (69 to 87)	80 (72 to 87)	73 (65 to 84)	**<0.01**
Baseline ScvO_2 _(%)	77 (69 to 82)	78 (70 to 82)	73 (57 to 78)	**0.01**
Six-hour ScvO_2 _(%)	77 (71 to 82)	77 (72 to 82)	72 (60 to 79)	**0.01**
Laboratories				
Lactate (mmol/l)	4.1 (2.2 to 6.3)	3.8 (1.9 to 5.8)	4.4 (3.0 to 6.9)	**<0.01**
Blood culture positive	180 (32.4%)	99 (29.9%)	81 (36.0%)	0.25
Culture positive	191 (34.4%)	103 (31.1%)	88 (39.1%)	0.05
White blood count (10^3^/mm^3^)	13.4 (6.9 to 20.4)	13.4 (7.2 to 20.2)	13.3 (6.7 to 22.0)	0.92
Hemoglobin (g/dl)	11.6 (9.7 to 13.2)	11.5 (9.6 to 13.2)	11.6 (9.8 to 13.4)	0.80
Platelet count (x10^9^/l)	150.0 (68.0 to 246.0)	162.0 (70.0 to 254.0)	136.0 (67.0 to 219.0)	0.07
International Normalized Ratio	1.4 (1.2 to 1.7)	1.3 (1.1 to 1.5)	1.5 (1.2 to 1.8)	**<0.01**
Creatinine (mg/dl)	2.0 (1.3 to 3.1)	2.0 (1.2 to 2.9)	2.0 (1.4 to 3.1)	0.24
Total bilirubin (mg/dl)	1.1 (0.7 to 2.4)	1.0 (0.6 to 2.2)	1.4 (0.7 to 3.4)	**<0.01**
Albumin (g/dl)	3.0 (2.5 to 3.6)	3.1 (2.6 to 3.7)	2.8 (2.3 to 3.5)	**0.01**
PaO_2_/FiO_2 _ratio	163.0 (86.0 to 320.7)	251.1 (95.0 to 391.0)	125.5 (77.0 to 217.0)	**<0.01**
Source of infection				0.13
Pneumonia	233 (41.9%)	136 (41.1%)	97 (43.1%)	
Urinary tract infection	93 (16.7%)	64 (19.3%)	29 (12.9%)	
Intra-abdominal	108 (19.4%)	68 (20.5%)	40 (17.8%)	
Skin/cellulitis	33 (5.9%)	17 (5.1%)	16 (7.1%)	
Other/unknown	89 (16.0%)	46 (13.9%)	43 (19.1%)	

Patient characteristics during the entire study period in the lactate clearance group compared with no lactate clearance. Results reported as either the count (column %) or as the median (25th to 75th percentile). APACHE, Acute Physiology and Chronic Health Evaluation; CVP, central venous pressure; ED, emergency department; MAP, mean arterial pressure; SSC, Surviving Sepsis Campaign; ScvO_2_, central venous oxygen saturation. ^a^Norepinephrine, dopamine, phenylephrine, or vasopressin. ^b^Dopamine or dobutamine.

Patients having lactate clearance had all Primary SSC Bundle items completed significantly more than those patients without lactate clearance (Table 2). Patients with lactate clearance had lower APACHE II score (*P *<0.01) and baseline lactate (*P *<0.01). The lactate clearance group had lower CVP and MAP but higher ScvO_2 _at baseline compared with the patients without lactate clearance (*P *<0.05). Patients with lactate clearance were more in the team care model than the nonteam model (*P *< 0.01) and received more fluid resuscitation both in the ED and ICU (*P *<0.01), with a shorter length of stay in the ED (*P *<0.01). Patients with lactate clearance had mortality of 20.5% compared with 43.6% in those patients with no lactate clearance (*P *<0.01).

**Figure 1:**
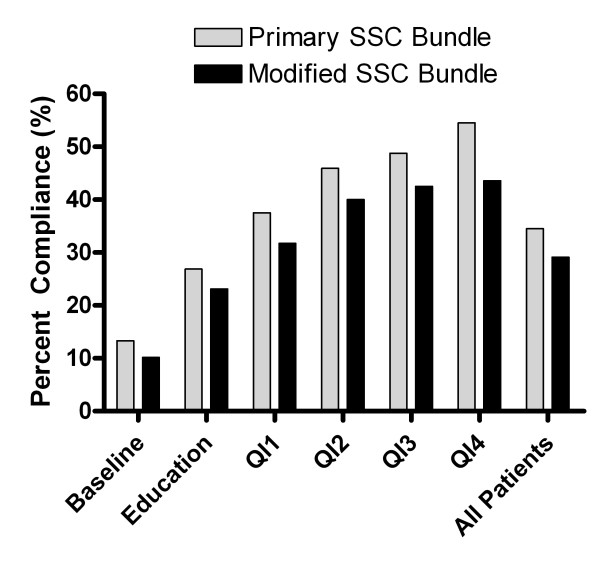
**Surviving Sepsis Campaign bundle completion**. Primary Surviving Sepsis Campaign (SSC) Bundle completion versus Modified SSC Bundle completion (includes lactate clearance) from baseline to the end of the study period. QI, quality improvement.

**Figure 2:**
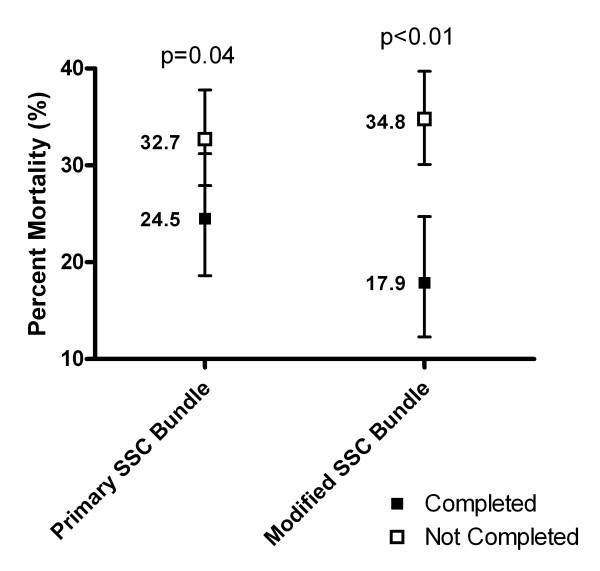
**Mortality differences for the Surviving Sepsis Campaign bundles**. Mortality differences for the Primary Surviving Sepsis Campaign (SSC) Bundle and the Modified SSC Bundle (includes lactate clearance). Patients with the Primary SSC Bundle completed compared with not completed had mortality of 24.5% (95% confidence interval = 18.6 to 31.2) versus 32.7% (95% confidence interval = 27.9 to 37.8). Patients with the Modified SSC Bundle completed compared with not completed had mortality of 17.9% (95% confidence interval = 12.3 to 24.7) versus 34.8% (95% confidence interval = 30.1 to 39.7). The ratio of the relative risk of death reduction for the Modified SSC Bundle compared with the Primary SSC Bundle was 1.94 (95% confidence interval = 1.45 to 39.1).

Completion of the Primary SSC Bundle over the six quartiles of implementation was 13.3, 26.9, 37.5, 45.9, 48.8, and 54.5%, respectively (*P *<0.01). The Modified SSC Bundle (including lactate clearance) was completed in 10.2, 23.1, 31.7, 40.0, 42.5, and 43.6% patients, respectively (*P *<0.01) (Table 3 and Figure 1). Patients with the Primary SSC Bundle completed compared with not completed had mortality of 24.5% (95% confidence interval = 18.6 to 31.2) versus 32.7% (95% confidence interval = 27.9 to 37.8) (*P *= 0.04). Patients with the Modified SSC Bundle completed compared with not completed had mortality of 17.9% (95% confidence interval = 12.3 to 24.7) versus 34.8% (95% confidence interval = 30.1 to 39.7) (*P *<0.01) (Figure 2). Completion of the Primary SSC Bundle resulted in a relative risk of death reduction (RRR) of 0.251 (95% confidence interval = 0.007 to 0.442), and completion of the Modified SSC Bundle had a relative risk of death reduction of 0.486 (95% confidence interval = 0.274 to 0.642). The ratio of the relative risk of death reduction for the Modified SSC Bundle compared with the Primary SSC Bundle was 1.94 (95% confidence interval = 1.45 to 39.1).

**Table 3:** Bundle compliance by implementation phases: baseline, education, and quality improvement phases 1 to 4

	All patients (*n *= 556)	Baseline (*n *= 128)	Education (*n *= 104)	QI1 (*n *= 104)	QI2 (*n *= 85)	QI3 (*n *= 80)	QI4 (*n *= 55)	*P *value
Lactate measured	488 (87.8)	99 (77.3)	78 (76.5)	100 (96.2)	84 (98.8)	77 (96.3)	50 (90.9)	**<0.01**
Blood cultures before antibiotics	514 (92.4)	101 (78.9)	97 (93.3)	99 (95.2)	82 (96.5)	80 (100.0)	55 (100.0)	**<0.01**
Antibiotics administered by 3 hours	555 (99.8)	128 (100.0)	103 (99.0)	104 (100.0)	85 (100.0)	80 (100.0)	55 (100.0)	0.50
Fluid bolus given	506 (91.0)	98 (76.6)	102 (98.1)	100 (96.2)	75 (88.2)	78 (97.5)	53 (96.4)	**<0.01**
Achieve CVP >8 mmHg by 6 hours	319 (57.4)	55 (43.0)	58 (55.8)	59 (56.7)	57 (67.1)	52 (65.0)	38 (69.1)	**<0.01**
Achieve MAP >65 mmHg by 6 hours	530 (95.3)	117 (92.1)	103 (100.0)	100 (96.2)	80 (94.1)	78 (97.5)	52 (94.5)	0.08
Achieve ScvO_2 _>70% by 6 hours	250 (45.0)	23 (18.0)	33 (31.7)	58 (55.8)	51 (60.0)	50 (62.5)	35 (63.6)	**<0.01**
Lactate clearance	331 (59.5)	68 (53.1)	43 (41.3)	63 (60.6)	60 (70.6)	61 (76.3)	36 (65.5)	**<0.01**
Primary SSC Bundle completion	192 (34.5)	17 (13.3)	28 (26.9)	39 (37.5)	39 (45.9)	39 (48.8)	30 (54.5)	**<0.01**
Modified SSC Bundle completion	162 (29.1)	13 (10.2)	24 (23.1)	33 (31.7)	34 (40.0)	34 (42.5)	24 (43.6)	**<0.01**

Results reported as count (column %). CVP, central venous pressure; MAP, mean arterial pressure; QI, quality improvement; ScvO_2_, central venous oxygen saturation; SSC, Surviving Sepsis Campaign.

Logistic regression models with sequential inclusion of each bundle item as the independent variable showed that the bundle items of fluid bolus given, achieve MAP >65 mmHg by 6 hours, and lactate clearance were independently associated with decreased mortality - having odds ratios (95% confidence interval) of 0.47 (0.23 to 0.96), 0.20 (0.07 to 0.55), and 0.32 (0.19 to 0.55), respectively, in the final model (Table 4).

**Table 4:** Logistic regression models with sequential inclusion of each bundle item and in-hospital mortality

	Model I		Model II		Model III		Model IV		Model V		Model VI		Model VII		Model VIII	
	
Bundle item	OR	95% CI	OR	95% CI	OR	95% CI	OR	95% CI	OR	95% CI	OR	95% CI	OR	95% CI	OR	95% CI
1. Lactate measured	1.96	(0.99 to 3.90)	2.03	(1.02 to 4.05)	2.03	(1.02 to 4.05)	2.27	(1.12 to 4.62)	2.38	(1.15 to 4.96)	2.67	(1.24 to 5.75)	2.63	(1.22 to 5.70)	4.06	(1.84 to 8.97)
2. Blood cultures before antibiotics			0.67	(0.29 to 1.54)	0.67	(0.29 to 1.54)	0.68	(0.29 to 1.59)	0.68	(0.29 to 1.59)	0.79	(0.33 to 1.90)	0.78	(0.33 to 1.89)	0.80	(0.33 to 1.94)
3. Antibiotics administered by 3 hours					1.14	(0.30 to 4.38)	1.06	(0.27 to 4.11)	1.09	(0.28 to 4.28)	1.18	(0.26 to 5.28)	1.20	(0.27 to 5.36)	1.35	(0.28 to 6.55)
4. Fluid bolus given							0.50	(0.25 to 0.99)	0.50	(0.25 to 0.99)	0.46	(0.23 to 0.93)	0.45	(0.22 to 0.92)	0.47	(0.23 to 0.96)
5. Achieve CVP >8 mmHg by 6 hours									0.89	(0.57 to 1.37)	1.05	(0.67 to 1.66)	1.01	(0.60 to 1.69)	1.21	(0.71 to 2.06)
6. Achieve MAP >65 mmHg by 6 hours											0.16	(0.06 to 0.44)	0.16	(0.06 to 0.43)	0.20	(0.07 to 0.55)
7. Achieve ScvO_2 _>70% by 6 hours													1.10	(0.65 to 1.87)	1.43	(0.82 to 2.51)
8. Lactate clearance															0.32	(0.19 to 0.55)
-2 log-likelihood	531.04		530.18		530.14		526.28		525.98		508.84		508.72		490.73	
Change in -2 log-likelihood (χ^2^)	-		-0.86		-0.04		-3.86		-0.30		-0.05		-0.12		-18.00	
Chi-square *P *value (df = 1)	-		0.35		0.84		0.05		0.58		0.82		0.73		< 0.01	

Logistic regression models with sequential inclusion of each bundle item, and odd ratios (OR) and 95% confidence interval (CI) for in-hospital mortality. The models are adjusted for age, gender, Acute Physiology and Chronic Health Evaluation II score, and mechanical ventilation. CVP, central venous pressure; df, degrees of freedom; MAP, mean arterial pressure; ScvO_2_, central venous oxygen saturation.

## Discussion

The rationale for implementing the severe sepsis care bundles has been that the compliance to the bundle would translate into better patient outcomes. Several studies showed that improvements in survival were related to the number of bundle interventions completed [16-18]. Recently, another study showed that mortality was significantly lower even if completion of the 6-hour resuscitation bundle was achieved later at 18 hours compared with not completing the bundle at all [19]. Despite a number of observational studies indicating protocol-driven EGDT and the severe sepsis bundle to be associated with reduced mortality, EGDT is underused even with formal bundle implementation in place [20]. Many potential barriers ranging from patient to clinician to organizational factors have been identified as stifling the more widespread compliance to the bundle. In the present study, we showed that implementation of the SSC resuscitation bundle in Asia was achievable with a compliance >50% after 1 year of quality improvement. Overall compliance was expectedly less throughout the study period with the addition of lactate clearance as a bundle item.

Our patients with optimal baseline CVP and ScvO_2 _contrast those of the original study examining EGDT. Optimizing preload remains a cornerstone for resuscitation of patients in shock; however, the use of CVP as a measure of fluid responsiveness has been challenged [21]. Evidence demonstrated a poor relationship between CVP and blood volume as well as its ability to predict a hemodynamic response to a fluid bolus. Irrespective of the debate and contrast to the low initial CVP observed in the original EGDT study, our patients did not require much fluid administration according to the target CVP recommended during the 6 hours of the resuscitation bundle. With respect to targeting ScvO_2_, the original EGDT study examined a patient population with initial ScvO_2 _<50% [15]. This finding has not been consistently observed in other studies, including ours. Another study showed that the number of severe sepsis patients with ScvO_2 _<50% was only 1%, with the rest of the patients having normal mean mixed venous oxygen saturation (SvO_2_) or ScvO_2 _values [22]. In fact, some studies have shown supranormal levels of ScvO_2_, which is probably due to the lack of effective tissue oxygen extraction that is seen in patients with sepsis [23].

Given the barriers and controversies in implementing EGDT, an approach utilizing noninvasive hemodynamic monitoring technology has been suggested [24,25]. Alternative to searching for the ideal technology, lactate monitoring for resolution of tissue hypoperfusion in septic shock seems practical and makes physiologic sense. A decreased in lactate (or lactate clearance) of ≥10% has been shown to be associated with improved 60-day survival [12,13]. Recently, lactate clearance >10% instead of ScvO_2 _>70% as an endpoint in the EGDT protocol was shown to result in similar amount of fluids, vasopressors, inotropes, blood transfusions, and in-hospital mortality [26]. Furthermore, a more aggressive approach at lactate clearance of 20% or higher every 2 hours, in addition to optimizing CVP, MAP, and ScvO_2 _during the first 8 hours, in mixed ICU patients resulted in decreased mortality [27]. One of our study investigators (HBN) previously included lactate clearance with EGDT in a 6-hour ED severe sepsis bundle and showed that lactate clearance was associated with decreased mortality [16]. In the present study we also found that the addition of lactate clearance to the SSC bundle was associated with greater improvement in outcome.

The suggestion that lactate clearance can be a replacement for the more dynamic endpoint of ScvO_2 _should be viewed with caution. ScvO_2 _as a surrogate to mixed venous oxygen saturation reflects the balance between oxygen delivery and oxygen consumption. Lactate elevation has been shown to be a surrogate for oxygen debt, or oxygen deficit accumulated over time [28,29]. Lactate clearance indicates the success of resuscitation in improving tissue perfusion and repayment of the oxygen debt. But the decrease in lactate also depends on the rate of production and metabolism in the muscle, kidney, and liver. Furthermore, normal lactate with persistent microcirculatory blood flow alterations can still suggest an unpaid oxygen debt [30].

The tenet of EGDT is to optimize oxygen delivery, resolving the oxygen transport imbalance, in the hope of improving tissue perfusion. Patients in the original EGDT study had significant hypovolemia, oxygen deficit, and hypoperfusion, with baseline ScvO_2 _<50% and lactate >7 mmol/l, indicating an oxygen-delivery-dependent state [15]. A low ScvO_2 _and high lactate have been shown to be independently associated with morbidity and mortality [31]. Achieving the ScvO_2 _>70% target at 6 hours was the only bundle element associated with reduction of in-hospital mortality [18]. The recent studies advocating lactate clearance as a therapeutic endpoint enrolled patients already having a normal ScvO_2 _at baseline [26,27]. In those patients, therefore, fluid resuscitation alone may be adequate to resolve tissue hypoperfusion, achieving lactate clearance, whereas patients with low initial ScvO_2 _will require blood transfusion and inotrope therapy to increase oxygen delivery [32].

In our study, we enrolled patients using the same inclusion criteria as the original EGDT study, but the targeted hemodynamic endpoints of CVP and ScvO_2 _were already optimal prior to therapy. We found that the fluid bolus, achieving MAP >65 mmHg, and lactate clearance were independent predictors of outcome. In our relative hemodynamically stable patients and in those patients in previous studies having optimal CVP and ScvO_2_, therefore, targeting MAP and lactate clearance may be adequate [22,33]. Our results would suggest that in these patients the requirement for specialized technology to monitor ScvO_2 _during the early treatment of septic shock might be avoided.

Our study had several limitations as it was a quality improvement effort aimed at improving the care delivery for severe sepsis patients, rather than a randomized examination of the effectiveness of the bundle and lactate clearance. The sample size was small for a multi-national study; however, we succeeded in showing that the resuscitation bundle can be implemented with a moderate level of compliance in developed and developing countries in Asia. We did not study the 24-hour management bundle including recombinant human activated protein C, since recombinant human activated protein C was not available in the study sites. Lactate clearance as a bundle item in our study was more of an observation of a resuscitation endpoint rather than part of a guided treatment protocol. We considered patients with initial lactate <2 mmol/l as achieving the lactate clearance criteria. Our results were similar when we excluded these patients from the analysis, however, and none of the patients with the initial normal lactate had increased values after successful initiation of the bundle (data not shown). Finally, our approach of adding lactate clearance to the bundle supports previous usage of monitoring lactate in addition to hemodynamic parameters of CVP, MAP, and ScvO_2 _[16,27].

## Conclusions

Our study demonstrated that adding lactate clearance as a component of the SSC resuscitation bundle resulted in a more significant mortality improvement, with an almost twofold increase in the relative risk of death reduction. Our data are consistent with previous studies and suggest that lactate clearance is a viable quality improvement goal for hospitals implementing the SSC guidelines to optimize outcome in patients with severe sepsis and septic shock.

## Key messages

• Implementation of the SSC resuscitation bundle at multiple hospitals in Asia significantly increased compliance over 18 months and was associated with improved outcome.

• Lactate clearance or the decrease in lactate after initiation of the SSC resuscitation bundle is associated with further improvement in outcome.

• In severe sepsis patients with hemodynamic stability, reflected by optimized baseline CVP and ScvO_2_, the bundle items of fluid bolus administration, achieving MAP >65 mmHg, and lactate clearance were independent predictors of outcome.

• Our study results do not imply that fluid resuscitation alone and the goals of MAP >65 mmHg and lactate clearance are adequate in those patients with evidence of significant organ hypoperfusion; that is, low ScvO_2 _and high lactate levels.

## Abbreviations

APACHE: Acute Physiology and Chronic Health Evaluation; CVP: central venous pressure; ED: emergency department; EGDT: early goal-directed therapy; MAP: mean arterial pressure; PaO_2_/FiO_2_: partial pressure of oxygen/fraction of inspired oxygen; ScvO_2_: central venous oxygen saturation; SSC: Surviving Sepsis Campaign.

## Competing interests

The investigator meetings for the present study were supported by Edwards Lifesciences, Asia Pacific, Singapore, in the form of administrative support and travel funding. HBN received funding support from Edwards Lifesciences, Asia Pacific, Singapore, to lead the study investigator meetings. The study sites received educational support from Edwards Lifesciences, Asia Pacific, Singapore, on the use of continuous ScvO_2 _monitoring. Edwards Lifesciences did not participate in the design of the study or the decision to submit this manuscript for publication.

## Authors' contributions

HBN provided leadership and oversight in all aspects of the study, including study design, data collection, data analysis, and manuscript preparation. WSK, PS, MM, C-HL, and SR contributed to the study design, data collection, and preparation of the manuscript. MB and AD contributed to the data analysis and preparation of the manuscript. All authors read and approved the final manuscript.
